# Cryptic Variation in the Human Mutation Rate

**DOI:** 10.1371/journal.pbio.1000027

**Published:** 2009-02-03

**Authors:** Alan Hodgkinson, Emmanuel Ladoukakis, Adam Eyre-Walker

**Affiliations:** Centre for the Study of Evolution, School of Life Sciences, University of Sussex, Brighton, United Kingdom; University of Edinburgh, United Kingdom

## Abstract

The mutation rate is known to vary between adjacent sites within the human genome as a consequence of context, the most well-studied example being the influence of CpG dinucelotides. We investigated whether there is additional variation by testing whether there is an excess of sites at which both humans and chimpanzees have a single-nucleotide polymorphism (SNP). We found a highly significant excess of such sites, and we demonstrated that this excess is not due to neighbouring nucleotide effects, ancestral polymorphism, or natural selection. We therefore infer that there is cryptic variation in the mutation rate. However, although this variation in the mutation rate is not associated with the adjacent nucleotides, we show that there are highly nonrandom patterns of nucleotides that extend ∼80 base pairs on either side of sites with coincident SNPs, suggesting that there are extensive and complex context effects. Finally, we estimate the level of variation needed to produce the excess of coincident SNPs and show that there is a similar, or higher, level of variation in the mutation rate associated with this cryptic process than there is associated with adjacent nucleotides, including the CpG effect. We conclude that there is substantial variation in the mutation that has, until now, been hidden from view.

## Author Summary

Understanding the process of mutation is important, not only mechanistically, but also because it has implications for the analysis of sequence evolution and population genetic inference. The mutation rate is known to differ between sites within the human genome. The most dramatic example of this is when a C is followed by G; both the C and G nucleotides have a rate of mutation that is between 10- and 20-fold higher than the rate at other sites. In addition, is it known that the mutation rate may be influenced by the nucleotides flanking the site. Here we show that there is also very substantial variation in the mutation rate that is not associated with the flanking nucleotides, or the CpG effect. Although this variation does not depend upon the adjacent nucleotides, there are nonrandom patterns of nucleotides surrounding sites that appear to be hypermutable, suggesting there are complex context effects that influence the mutation rate.

## Abstract 3

Substantial variation is seen in the rate of mutation among different sites in the human genome, which is not associated with mutable CpG sites or due to simple context effects.

## Introduction

The mutation rate is thought to vary across the human genome on several different scales. At the chromosomal level, the Y chromosome evolves faster than the autosomes, which evolve faster than the X chromosome [1,2]. This is thought to be due to males having a higher mutation rate than females. The autosomes also appear to differ in their rates of mutation for reasons that are unclear [3,4]. At the next level down, there appears to be variation in the mutation rate over a scale of several hundred kilobases [4,5], another pattern that remains unexplained. However, the most dramatic variation in the mutation rate is observed over fine scales in which adjacent sites can have very different mutation rates. In the nuclear genome, this variation has been shown to be associated with context, the best-known example being the CpG dinucleotide in mammals. CpG dinucleotides are generally methylated in mammals and since methyl-cytosine is unstable, this leads to a high rate of C→T and G→A transitions at these sites, which is about 10- to 20-fold higher than at other sites [6,7]. However, the CpG effect is not the only source of fine-scale variation in the mutation rate; the rate of mutation appears to vary by about 2- or 3-fold as a function of other adjacent nucleotides [8–11].

Although variation in the mutation rate has been well-characterised in terms of adjacent nucleotides [8,9,11], it is possible that there is other variation in the mutation that is associated with either distant or complex context effects, which has hitherto escaped detection. We investigated this question by testing whether human and chimpanzee single nucleotide polymorphisms (SNPs) occur at orthologous sites in the genome. If there is variation in the mutation rate, we expect to see an excess of sites at which both humans and chimpanzees have a SNP.

## Results

### Excess of Coincident SNPs

**Figure 1:**
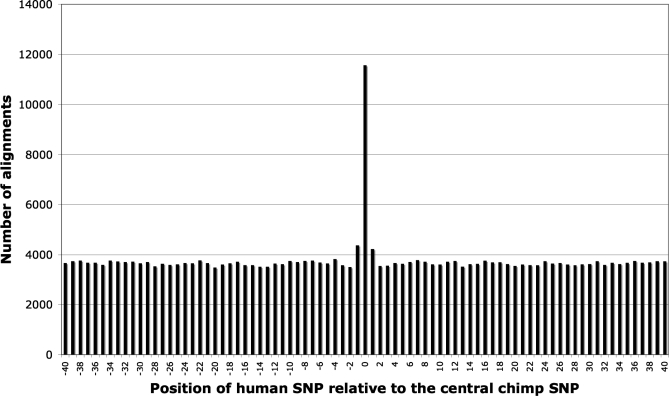
The Number of Human SNPs at Each Site of the Human–Chimpanzee Alignments Used in the Analysis

To investigate whether human and chimpanzee SNPs tend to occur at the same sites in the genome, we BLASTed all chimpanzee SNPs against a dataset of human SNPs. This yielded a dataset of 309,158 alignments of 81 base pairs (bp) with the chimpanzee SNP in the central position and a human SNP elsewhere within the alignment. Of these alignments, 11,571 have the human and chimpanzee SNP at the same position (Figure 1); we refer to these as coincident SNPs. This number of coincident SNPs is much greater than the 3,817 we would expect if the human SNPs were distributed at random across the alignment, and also much greater than the 6,592 we would expect taking into account the influence of the adjacent nucleotides on the mutation rate, henceforth known as “simple” context effects. The observed excess of coincident SNPs is significantly greater than the expected number (ratio of observed over expected with simple context effects = 1.76, with a standard error of 0.02, *p* < 0.0001 under the null hypothesis that the ratio is 1). This excess is not due to our inability to correct for CpG effects; if we remove CpG dinucleotides from the analysis, we observe 5,028 coincident SNPs but would only expect 2,533 taking into account simple context effects (ratio = 1.98 (0.03); *p* < 0.0001). If we look at the pattern of coincident SNPs, it is evident that almost all the excess is due to the same SNP being present in both humans and chimpanzees, with A-T/A-T SNPs being dramatically over-represented (Table 1; see Table S1 for the analysis with CpG sites removed).

**Table 1:**
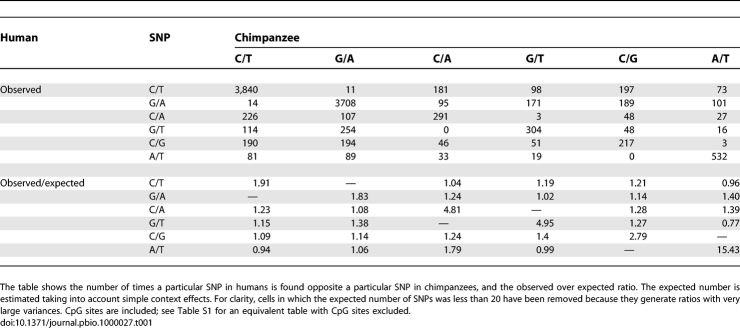
The Pattern of Coincident SNPs

Although the excess of coincident SNPs is consistent with variation in the mutation rate that is not associated with simple context, there are several other explanations that warrant consideration.

### Strand Asymmetry

In correcting for simple context effects, we have also made two assumptions; we have assumed that the pattern of mutation is the same on the two strands of the DNA duplex, and we have assumed that context effects are the same across the genome. As a consequence of these assumptions, we could be underestimating the expected number of coincident SNPs. For example, let us imagine that the triplet AAA has a high mutation rate on one strand, say the transcribed strand, and a low mutation rate on the other strand, but that the pattern is the opposite for the triplet CCC (note that when we refer to the mutation of a triplet, we are referring to the mutation rate of the central nucleotide). Because the relative mutation rates of AAA and CCC depend on which strand we are considering, we would tend to underestimate the expected number of coincident SNPs.

The pattern of mutation is known to differ between the two DNA strands in a manner that depends on transcription [12,13]. However, what is important for our analysis is whether the relative mutation rates of the triplets differ between strands; it is the relative, rather than the absolute rate, that matters, because for each alignment we calculate the chance of a coincident SNP relative to the chance that the human SNP occurs at one of the other triplets in the sequence. To investigate this, we estimated the mutation rate of the central nucleotide in each triplet for a set of human genes for which we knew the direction of transcription; we also considered a subset of these genes known to be expressed in the testis.

In agreement with Green et al. [12], we observe a 25% excess of A→G transitions over T→C transitions; however, we did not observe an excess of G→A transitions over C→T transitions, even in our testis-expressed genes. Crucially for our analysis, the mutation rate of each triplet is highly correlated to its reverse-compliment triplet for all genes (Pearson correlation coefficient *r* = 1.00 for all triplets, *r* = 0.85 without triplets containing CpGs; Figure S2A) and for genes expressed in the testes (*r* = 0.99 for all triplets, *r* = 0.75 without triplets containing CpGs; Figure S2B); genes expressed in the testes are expressed in the male germ-line, where any strand asymmetry in the pattern of mutation will have an evolutionary effect. It therefore seems unlikely that strand asymmetry in the pattern of mutation is leading to an underestimate of the expected number of coincident SNPs.

### Patterns of Mutation

The excess of coincident SNPs could also be due to variation in the pattern of mutation across the genome for reasons similar to those given for strand asymmetry; if the relative rate at which each triplet mutates differs between genomic regions, then we will underestimate the expected number of coincident SNPs. Since such variation in the pattern of mutation might be expected to generate differences in base composition, we divided our dataset of alignments according to their GC content and estimated the mutation rate of the central nucleotide in each triplet in the chimpanzee sequence using the human sequence to infer the ancestral sequence. The relative rates of mutation inferred from the sequences in the upper and low GC content quartiles are highly correlated to each other (*r* = 0.99 using all triplets; *r* = 0.88 excluding triplets involving CpGs; Figure S3), which suggests that triplets that are highly mutable in high–GC content sequences also tend to be highly mutable in the low–GC content sequences. It therefore seems unlikely that we are underestimating the expected number of coincident SNPs because of variation in the pattern of mutation. As expected, we find a significant excess of coincident SNPs in both the upper and lower GC quartile datasets, although the excess of coincident SNPs appears to be slightly stronger in GC-poor DNA (Table S2).

### Ancestral Polymorphism

The excess of coincident SNPs could be due to inheritance, in humans and chimpanzees, of polymorphisms that were present in their last common ancestor. Two lines of evidence suggest that this is not the case. First, we repeated the analysis using human and macaque SNPs. Since these two species diverged more than 23–34 million years ago (Mya) [14], as opposed to the 6–10 My that separates human and chimp [14], one would expect very few polymorphisms to be shared between human and macaque. However, in this dataset we also see a significant excess of coincident SNPs whether we consider all sites (ratio = 1.64 (0.19); *p* < 0.001) or non-CpG sites (1.51 (0.26); and *p* < 0.05). Second, the pattern of coincident SNPs (Table 1) is inconsistent with ancestral polymorphism. All four of the possible transversion SNPs are approximately equally common amongst SNPs in general (proportion of transversions amongst human SNPs: G/T = 0.092, C/A = 0.091, C/G = 0.088, A/T = 0.075; transitions: C/T = 0.33, G/A = 0.33). We would therefore expect a G-C SNP in chimps to be coincident with a G-C SNP in humans approximately equally often as an A-T SNP in humans is coincident with an A-T SNP in chimps. However, we see distinct biases, with coincident A-T/A-T SNPs being much more common than the other transversions.

### Natural Selection

It is also possible for the apparent excess of coincident SNPs to be due to selection; if some regions of the genome are under selection, then we expect them to have a low density of SNPs, because many SNPs will be removed as they are deleterious. As a consequence, SNPs will be clustered between these regions, causing an apparent excess of coincident SNPs. This seems an unlikely explanation, since the vast majority of our data is intergenic and intronic (98% and 99% of the human and chimpanzee SNPs in our BLAST databases, respectively), and although selection is known to act within these regions, it is thought to only affect a small percentage of sites [15–17]. Furthermore, if selection was causing an excess of coincident SNPs, we would expect SNPs to be clustered generally, but this is not observed (Figure 1 and Figure S1). There is a small excess of human SNPs adjacent to the chimpanzee SNP, but this is a consequence of CpG effects—the chimpanzee SNP is disproportionately likely to occur within a CpG, which means that a human SNP is also likely to occur at the same site, or at an adjacent site. If we remove CpGs, this slight excess of adjacent SNPs disappears (Figure S1). Otherwise there is no tendency for SNPs to cluster.

### Other Context Effects

It therefore seems that the excess of coincident SNPs is a consequence of variation in the mutation rate that is not associated with simple context effects, variation in these context effects between strands or regions of the genome, or natural selection. The question therefore arises whether the variation in the mutation rate is associated with other contexts that are distant from the target site, degenerate in nature, or sufficiently complex to be difficult to discern. It should be noted that simple context effects beyond the adjacent nucleotides (e.g., 1 bp removed from the target site) are not responsible for the excess. Although these effects exist [11], they are much smaller than those of adjacent nucleotides, which themselves have a relatively modest effect if we remove CpGs; e.g., the expected number of non–CpG coincident SNPs is 2,115 if we ignore adjacent nucleotide effects, and it is 2,533 if we include these effects.

**Figure 2:**
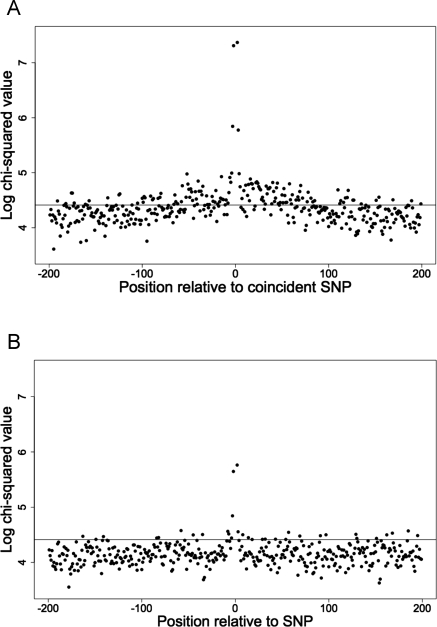
Heterogeneity in Triplet Frequencies This figure gives the log value from a chi-square test of heterogeneity of triplet frequencies at each site of the human–chimpanzee alignment versus the average triplet frequencies across the whole alignment for (A) alignments containing a coincident SNP, and (B) alignments without a coincident SNP, but with a chimpanzee SNP at the central position. The line marks the point above which 5% of the chi-square values are expected to fall by chance alone. The chi-square values are not given for the central three sites because the presence of the chimpanzee SNP in the centre of the alignment means that triplets cannot be counted at positions 0, +1, and −1.

To investigate whether there are other, more complex context effects, we tabulated the frequency of each triplet at each site in the alignments containing coincident SNPs, and a similar-sized dataset of alignments with noncoincident SNPs. Surprisingly, we found significant heterogeneity in triplet frequencies that extends to about 80 bp on either side of the coincident SNP (Figure 2A); i.e., the relative frequencies of the triplets at sites close to the coincident SNP are different from the average across the alignments. In contrast, if we consider alignments without a coincident SNP, but with a chimpanzee SNP, we only see significant heterogeneity in triplet frequencies within 10 bp of either side of the SNP (Figure 2B). Despite the heterogeneity in triplet frequencies surrounding a coincident SNP, we could discern very few patterns in the triplets that are over- or under-represented. The only conspicuous pattern is an excess of TTT triplets upstream and AAA triplets downstream of coincident SNPs. However this seems to explain little of the overall excess of coincident SNPs. If we repeat the analysis but remove all cases in which there is a run of three or more nucleotides, of any type, with or without SNPs within them, then from our alignments we find 8,536 alignments with a coincident SNP versus an expected number of 4,434, taking into account simple context effects (ratio = 1.93 (0.02); *p* < 0.0001). Considering pentamers, rather than triplets, also fails to reveal any context that is associated with coincident SNPs, except for the α-polymerase pause site motif, TG(A/G)(A/G)(G/T)(A/C), which has been suggested as a hypermutable motif [18,19]. However, we only observe an excess of α-polymerase pause sites immediately downstream of coincident SNPs, and the total number of coincident SNPs explained by this motif is trivial (2.2%).

### Quantification

To quantify the level of cryptic variation in the mutation rate, we fit two models to the ratio of the observed number of coincident SNPs over the number expected with simple context effects. In the first model, we assumed that the variation in the mutation rate was log-normally distributed; in the second, we assumed that there were two types of sites—normal and hypermutable. These models give qualitatively similar estimates of the variation, so we only discuss the log-normal model in detail, because this is a model with a single parameter (details of the two-rate model are given in Text S1). Because our method for controlling for simple context effects tends to underestimate the expected number of coincident SNPs when we have CpG sites, we concentrate on non-CpG sites. We fit two sub-models to our data. In the first, we assume that the mutation rate of a site is invariant in both humans and chimpanzees. Under this “static” model, we estimate the shape parameter of the log-normal to be 0.83 (95% confidence intervals (CIs) of 0.81, 0.84) for non-CpG sites. However, this model may not be realistic, since we might expect sites with high mutation rates to destroy themselves; e.g., if a site has a high rate of C→T mutation, then it will rapidly become fixed for T and therefore become nonhypermutable. We therefore also fit a model in which the time a site remains at a certain mutation rate depends upon that mutation rate, assuming an average divergence between humans and chimpanzees of 0.92% for non-CpG sites [20]. Under this model, we estimate slightly higher levels of cryptic variation: we estimated the shape parameter to be 0.85 (0.83, 0.87)—higher shape parameters mean more variation. The level of variation that these distributions represent is considerable; with a shape parameter of 0.85 the fastest 5% of sites mutate at least 16.4-fold faster than the slowest 5% of sites. This level of variation in the mutation rate is greater than the variation associated with simple context: the variance due to simple context, including CpGs, is 0.59, whereas the variance due to cryptic variation at non-CpG sites is 1.05. However, this large difference in variance might be due to the model. If we consider a simple two-rate model in which sites are either hypermutable or normal, and constrain the proportion of hypermutable sites to be 2%, which is the proportion of sites that are involved in CpGs in the human genome [21], then we estimate that hypermutable sites would have to mutate 9.3-fold faster than normal sites to explain the excess of coincident SNPs. This is similar to 10–20-fold higher rate that CpGs mutate [9,20].

## Discussion

We have shown that there is an excess of sites that have a SNP in both the human and chimpanzee genomes. We demonstrated that this is not due to neighbouring nucleotide effects, shared ancestral polymorphism, or natural selection. It therefore seems that this excess is due to variation in the mutation rate that is not associated with simple context effects and is cryptic in nature. We also show that triplet frequencies surrounding sites with coincident SNPs are highly nonrandom, but we have been unable to discern any specific motifs in these regions. This suggests that there are probably complex context effects that extend some distance from the site they effect. Furthermore, we show that there has to be considerable variation in the mutation rate to explain the observed excess of coincident SNPs.

The presence of such cryptic variation in the mutation rate is perhaps not surprising given the evidence that some sites in the human mitochondrial genome are hypermutable. Hypermutation had long been suspected based on the excess of homoplasies in human mitochondrial DNA (mtDNA) phylogenies (e.g., see [22]) and although such an excess could be due to hypermutation or recombination [23], two recent analyses have provided convincing evidence that the excess is due to hypermutation. Stoneking [24] showed that mitochondrial mutations in human pedigrees tend to occur at sites that have high levels of homoplasy, and Galtier et al. [25] have recently shown that synonymous mitochondrial SNPs tend to occur at the same positions in different species.

However, although many of the hot spots in mtDNA appear to be due to strand slippage–type mutational mechanisms [26,27], this does not appear to be case for the cryptic variation in the mutation rate in nuclear DNA that we describe here. There are two slippage mechanisms that can operate: template strand and primer strand dislocation. Template strand dislocation is controlled for in our simple context analysis, and primer strand dislocation is controlled for in the analysis of homonucleotide runs.

It has also been shown recently that the mutation rate is elevated close to insertion and deletion mutations in the nuclear genomes of several eukaryotes, including humans [28]. However, it seems unlikely that this process is generating the excess of coincident SNPs. Indels appear to increase the rate of mutation but not at specific sites; rather the mutation rate is elevated close to an indel and this elevation in the mutation rate declines over several hundred nucleotides. This would manifest itself as general tendency for SNPs to cluster, which we do not observe (Figure 1 and Figure S1); we only observe a large excess of coincident SNPs and a small excess of adjacent SNPs. Furthermore, humans and chimpanzees would both have to have segregating indels in the same locality to generate an excess of coincident SNPs.

Over the last few years, DNA sequence analysis has revealed that the mutation process is highly complex, varying between different parts of the genome and between different sites. Unfortunately we do not yet understand many of these patterns.

## Materials and Methods

### Data.

We downloaded human and chimpanzee SNPs from dbSNP build 126. Dividing the data into chromosomes, we BLASTed each chimpanzee SNP, along with 50 bp of flanking DNA on either side of the SNP, against a database of human SNPs. We set the BLAST parameters as follows; e-value = 1 × 10^−30^, mismatch score = −1, and simple sequence filter off. We retained those alignments, which were 101 bp in length, and in which the human or chimpanzee sequence showed identity at 96 sites if the SNPs were coincident, or 94 sites if they were not coincident. We adjusted the number of matches required to control for the fact that if the SNPs are not coincident, then there must be two extra mismatches. We randomly chose one alignment if a chimpanzee SNP matched more than one human SNP at the levels of identity we set; we obtained very similar results removing these cases from the analysis. The alignments were trimmed to 40 bp on either side of the central chimpanzee SNP because there is a slight bias away from finding human SNPs at the edges of the chimpanzee query sequence. This bias occurs because SNPs, being classed as mismatches, tend to cause BLAST to prematurely terminate the alignment. To perform the analysis of triplet frequencies, we downloaded an extended flanking sequence for the chimpanzee SNPs analysed.

The macaque SNPs were kindly provided by Dr. Ripan Malhi [29]. We repeated the analysis as we did for chimpanzee but we relaxed the criteria used to identify orthologous human sequences containing SNPs to 86 matches if there was a coincident SNP, and 84 if there was not, with the e-value adjusted to allow this level of similarity to be found.

Sites were designated as CpG if the site, or any of the SNPs at the site, would yield a CpG dinucleotide.

### Estimating the expected number of coincident SNPs.

We estimated the expected number of coincident SNPs, taking into account the effects of adjacent nucleotides on the rate of mutation, what we term “simple” context effects, as follows. Our data consist of a set of alignments in which we have both a human and a chimpanzee SNP. We start by tabulating the numbers of each triplet, *n_xyz_*, where *x*, *y*, and *z* can be T, C, A, or G, in the chimpanzee sequence in the alignments, along with the number of chimp triplets that have a human SNP opposite the central nucleotide, *n_xyz.Hsnp_*. From these, we can estimate the probability of observing a human SNP opposite a chimpanzee triplet in our alignments: *p_xyz_ = n_xyz.Hsnp_ / n_xyz_*. We can also calculate the frequency of each triplet in the chimpanzee sequences: *f_xyz_ = n_xyz_/*Σ*n_xyz_* To calculate the probability that the human and chimpanzee SNPs are coincident, we need to take into account that there are two alleles in the chimpanzee SNPs, and the triplets they are a part of will have different probabilities of having a human SNP opposite them. If we knew the relative frequencies of the chimpanzee alleles, we could calculate the chance of a coincident SNP as *g_y_ p_xyz_* + (1– *g_y_*)*p_xy'z_* where *y* and *y'* are the two chimpanzee alleles and *g_y_* is the frequency of the *y* allele. However, we do not have allele frequency information, so we estimated the relative probabilities of each of the two ancestral states for the chimpanzee SNP, since the ancestral allele is likely to be at a higher frequency in the population. For example, let us imagine we have a CYC SNP—i.e., a Y SNP surrounded by C on both sides. The ancestral triplet could have been CCC or CTC. The probability that the SNP was generated from a CCC can be estimated as *m*_CCC_ *= f*_CCC_ *r*_CCC_/(*f*_CCC_ *r*_CCC_ + *f*_CTC_ *r*_CTC_) where *r_xyz_* is the rate at which triplet XYZ generates a SNP in the central position of the triplet. We estimate *r_xyz_* by orienting the chimp SNPs using the human sequence, excluding coincident SNPs and SNPs for which the human nucleotide is different to both chimp alleles; let *s_xyz.Csnp_* be the number of chimp triplets that are inferred to have generated a SNP, then *r_xyz_* = *s_xyz,Csnp_/n_xyz_*. The expected number of coincident SNPs in each alignment is then, using the above example, (*m*_CCC_ *p*_CCC_ + *m*_CTC_ *p*_CTC_)/Σ*p_xyz_*, where the summation is across all the triplets in the alignment. The total number of expected coincident SNPs was simply the sum across alignments.

We used two methods to calculate the standard error for the ratio of the observed number of coincident SNPs over the expected number: we bootstrapped the data by alignment and then summed the observed and expected values across the bootstrapped datasets. However, it turned out that this was very closely approximated by assuming that the observed number of coincident SNPs was Poisson distributed and the expected value was known with no error; these are the standard errors we present.

### Simulations.

We performed a number of simulations to check that the BLAST analysis was not biased and that our method to estimate the number of coincident SNPs under simple context effects worked well. In each simulation, we evolved human genomic sequences under a mutation pattern, in which the mutation rate depended on the adjacent nucleotides, to generate a simulated human and chimpanzee sequence. Into these we introduced SNPs according to the same mutation pattern at the density found in dbSNP—one SNP every 266 bp in humans and every 2,128 bp in chimp. We then constructed a BLAST database of ∼140,000 human SNPs with 100 bp of flanking DNA sequence, and a query dataset of ∼18,000 chimpanzee SNPs with 50 bp of flanking DNA. We ran the BLAST analysis and analysed the output exactly as we had with the real data. We ran simulations in which we had no mutation bias and datasets in which the mutation rate of all triplets was the same except for triplets containing CpGs, which had a mutation rate 10, 15, or 20 times the background rate. We ran a set of simulations in which we had 0%, 1%, and 2% divergence. Our method works well at all divergences and under all mutation patterns, except when the CpG rate is very high, where the method tends to underestimate the expected number of coincident SNPs (Table S3). Surprisingly, the method tends to slightly overestimate the expected number of coincident SNPs when CpG sites are removed for reasons that are not clear.

### Strand asymmetry.

To investigate strand asymmetry, we estimated the mutation rate of the central nucleotide in each triplet by tabulating the number of times each triplet contained a SNP. The direction of mutation was inferred from the frequency; i.e., the minority allele was judged to be the new mutation. We inferred mutation rates across 964 human genes from the Seattle SNPs [30] and Environmental Genome Projects [31]. To investigate which of these genes are expressed in the male germ line, we downloaded gene expression data from the human testis from the study of Ge et al. [32]. We obtained raw CEL files of gene expression levels from the NCBI Gene Expression Omnibus database (http://www.ncbi.nlm.nih.gov/projects/geo/). We normalized the results from the mouse and rat arrays separately using the RMA algorithm [33] as implemented in Bioconductor [34]. We judged a gene to be expressed within the testis if its expression was above 200 [35].

### Log-normal model.

We estimated the variation in the mutation rate as follows. We start by assuming there is no divergence between humans and chimpanzees so a hypermutable site in humans will also be hypermutable in chimpanzees. Let the average probability of detecting a SNP at a site in humans and chimpanzees be μ*_h_* and μ*_c_*, respectively; if μ*_h_* and μ*_c_* are small, the probability at a particular site will be γμ*_h_* and γμ*_c_*, where γ is the relative rate of mutation. Let us assume that γ takes some distribution *D*(γ) which has a mean of one. The expected number of coincident SNPs is


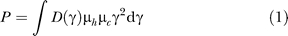


If there is no variation in the mutation rate then this reduces to


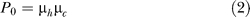


such that the ratio of the number of coincident SNPs, over the number expected with no variation, is


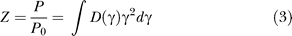


an equation which only depends upon the distribution of γ. We assume that γ is either log-normally distributed, or that it has a two state distribution in which sites can either be hypermutable or normal (see Protocol S1). We estimate the parameters of the distribution of γ by considering the ratio of the observed number of SNPs over the number expected with simple context effects (i.e., the number expected without cryptic variation in the mutation rate).

This model is unrealistic, because we assume that a site does not change its mutation rate; however, hypermutable sites are more likely to change, and this may lead them to become nonhypermutable. Under the log-normal model, we assume that once a site changes, its mutation rate is drawn randomly from the log-normal distribution. Let *v* be the average rate of mutation per unit time in both humans and chimpanzees. Consider a site, in the ancestor of humans and chimpanzees, that currently has a mutation rate *v*γ. The probability that the site will remain unchanged along both the human and chimpanzee lineage is


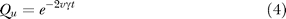


where *t* is the time since humans and chimpanzees diverged. The probability that such a site will produce a coincident SNP is


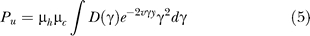


If the site changes in one of the lineages, then the mutation rates in the two lineages become independent of one another; since the mean of a product is the product of the means, when two random variables are independent, the probability of a coincident SNP at a site which has undergone at least one substitution is


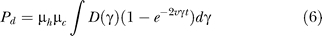


The expected number of SNPs with no variation in the mutation rate is still *P*_0_, as given by Equation 2, so we can write the ratio of the expected number of coincident SNPs with variation over the expected number without variation in the mutation rate as


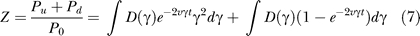


This equation depends on the compound parameter 2*vt*, which is the average divergence between humans and chimpanzees and the distribution of γ. Since we set the average of the log-normal distribution to one, we need only find the shape parameter of the log-normal distribution.

To estimate the variance associated with simple context effects, we calculated the mutation rate of each triplet as above, when correcting simple context effects. We then scaled the mutation rates so the mean across triplets, taking into account their frequencies in the genome, had a mean of one. We then calculated the variance. This can be compared directly to the variance of the log-normal distribution which we had also constrained to have a mean of one. We weighted the variance estimates from the CpG and non-CpG sites by the relative frequency of the sites.

## Supporting Information

Figure S1 The Number of Human SNPs at Each Site of the Human–Chimpanzee Alignments Used in the Analysis Excluding CpG Sites The slight deficit of human SNPs adjacent to the chimpanzee is caused by the adjacent sites being more likely to be inferred to be within a CpG because the chimp SNP might contain either C or G. For example, if the human SNP at +1 is G/A and the chimp SNP is C/G, this would be called a potential CpG site and excluded.
(56 KB PDF)

Figure S2 The Rate of Mutation for Each Triplet and Its Reverse Complement (A) All genes and (B) genes expressed in the testes.
(35 KB PDF)

Figure S3 The Rate of Mutation for Each Triplet in the GC-Rich Alignments (*x*-Axis) Versus the Rate of Mutation in the GC-Poor Alignments (*y*-Axis) (28 KB PDF)

Table S1 The Pattern of Coincident SNPs at Non-CpG Sites The table shows the number of times a particular SNP in humans is found opposite a particular SNP in chimpanzees, and the observed-over-expected ratio excluding CpG sites. Note that some of the observed values are greater than when we included CpG dinucleotides. This is because we re-ran the analysis and when a chimp SNP had matched multiple human sequences, we chose a sequence in which the human SNP was not involved in a CpG. Ratios are omitted when the expected value was less than 20.
(61 KB DOC)

Table S2 The Observed and Expected Numbers of Coincident SNPs in the Alignments with High or Low GC Content (27 KB DOC)

Table S3 The Observed and Expected Number of Coincident SNPs from Simulations Run with Different Levels of CpG Hypermutation and Divergence (51 KB DOC)

Table S4 The Relative Rates of Mutation at Normal and Hypermutable Sites in the Two-Rate Model (27 KB DOC)

Text S1 Supporting Methods (28 KB PDF)

## Abbreviations

Mya: million years ago
SNP: single-nucleotide polymorphism
