# Supplementary material for: Cryptic Variation in the Human Mutation Rate
Source: PLoS Biol. 2009 Feb 3;7(2):e1000027. doi: 10.1371/journal.pbio.1000027 (PMC2634788; doi:10.1371/journal.pbio.1000027)
Supplement: Supplementary file 1 [file pbio.1000027.sg001.pdf]

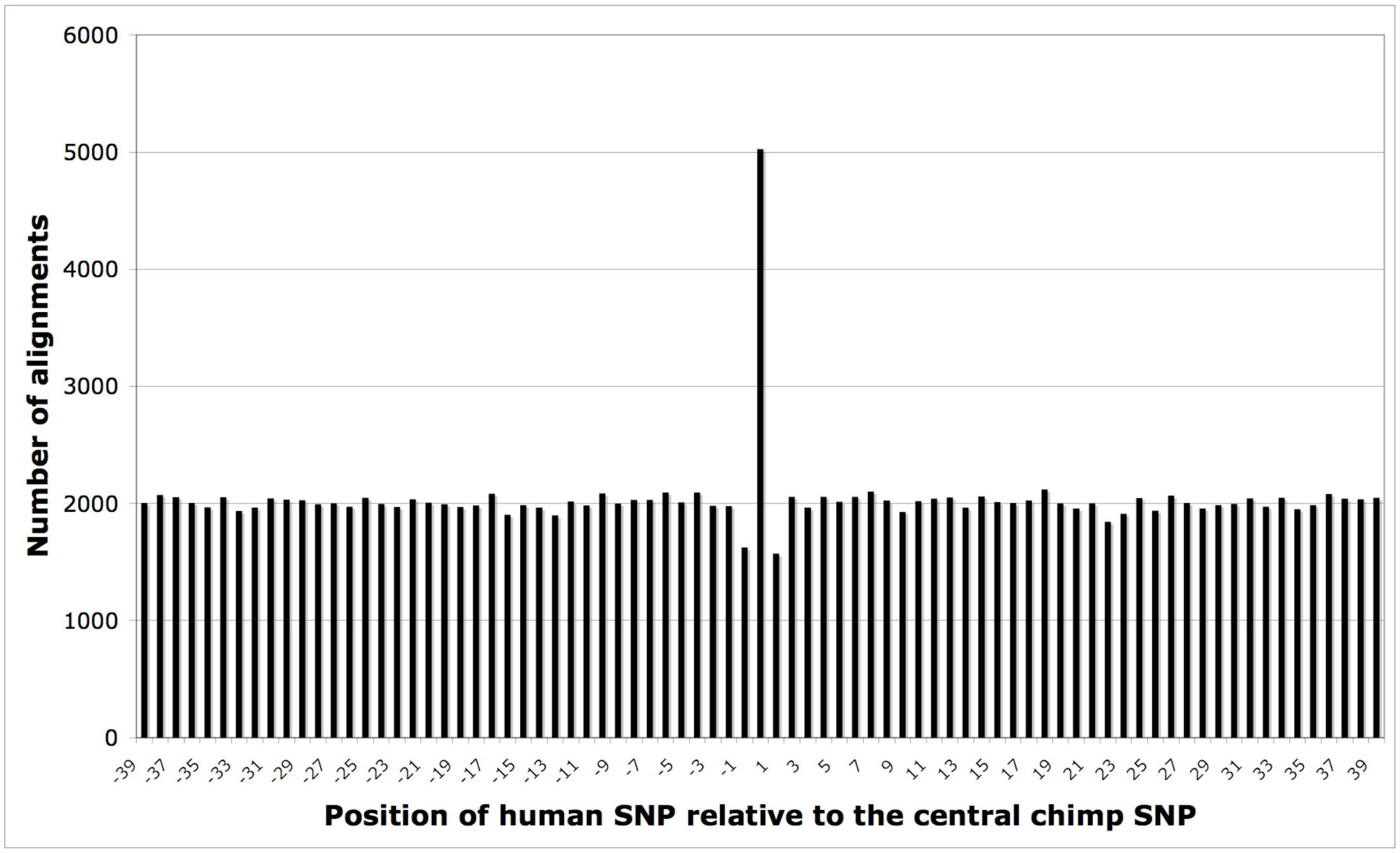

Number of alignments
0
1000
2000
3000
4000
5000
6000
-39
-37
-35
-33
-31
-29
-27
-25
-23
-21
-19
-17
-15
-13
-11
-9
-7
-5
-3
-1
1
3
5
7
9
11
13
15
17
19
21
23
25
27
29
31
33
35
37
39
Position of human SNP relative to the central chimp SNP
