# Supplementary material for: Cryptic Variation in the Human Mutation Rate
Source: PLoS Biol. 2009 Feb 3;7(2):e1000027. doi: 10.1371/journal.pbio.1000027 (PMC2634788; doi:10.1371/journal.pbio.1000027)
Supplement: Supplementary file 2 [file pbio.1000027.sg002.pdf]

2a)

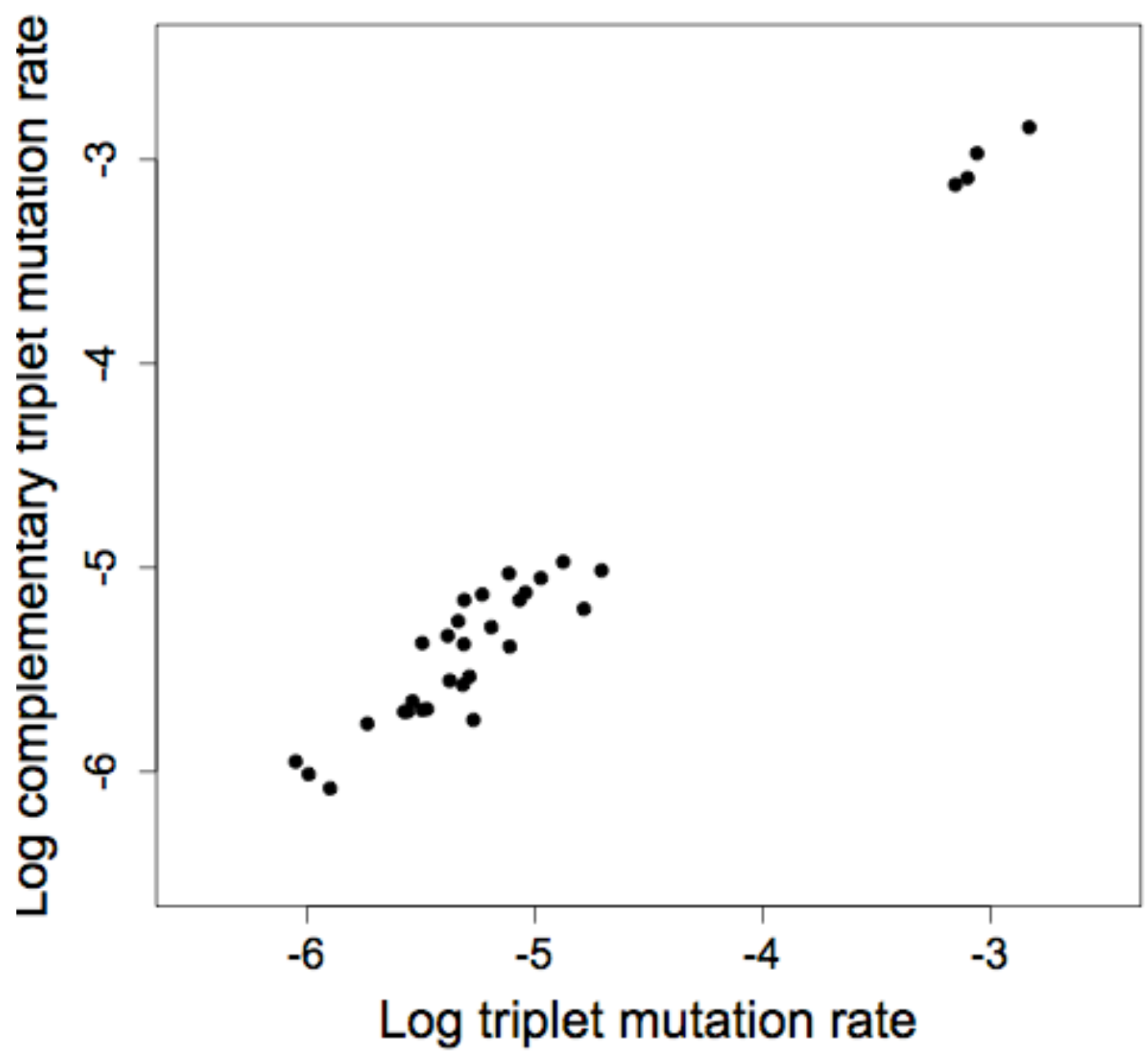
Log complementary triplet mutation rate
-3
-4
-5
-6
-6
-5
-4
-3
Log triplet mutation rate

2b)

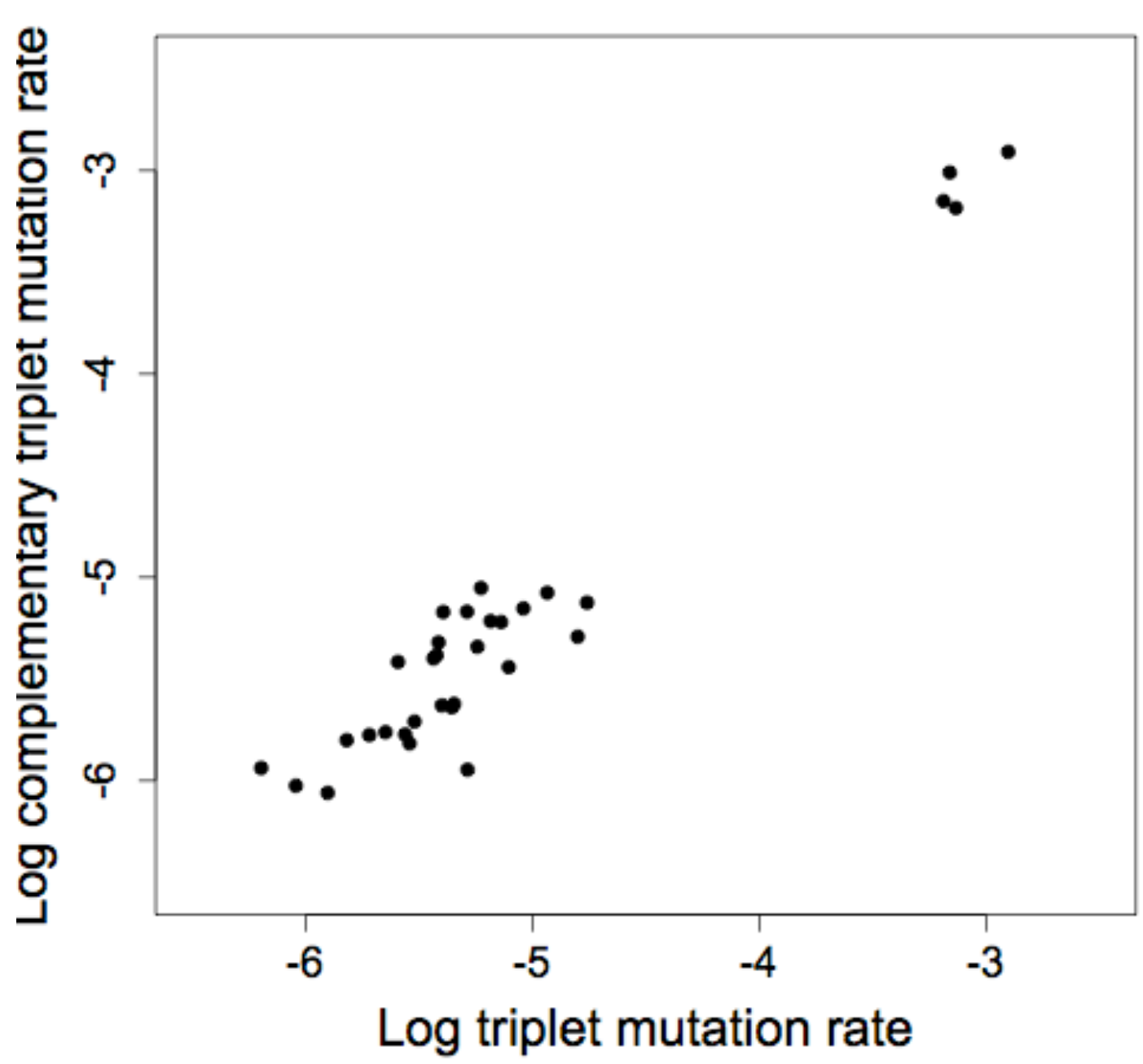
Log complementary triplet mutation rate
-3
-4
-5
-6
-6
-5
-4
-3
Log triplet mutation rate
