# Supplementary material for: Cryptic Variation in the Human Mutation Rate
Source: PLoS Biol. 2009 Feb 3;7(2):e1000027. doi: 10.1371/journal.pbio.1000027 (PMC2634788; doi:10.1371/journal.pbio.1000027)
Supplement: Supplementary file 3 [file pbio.1000027.sg003.pdf]

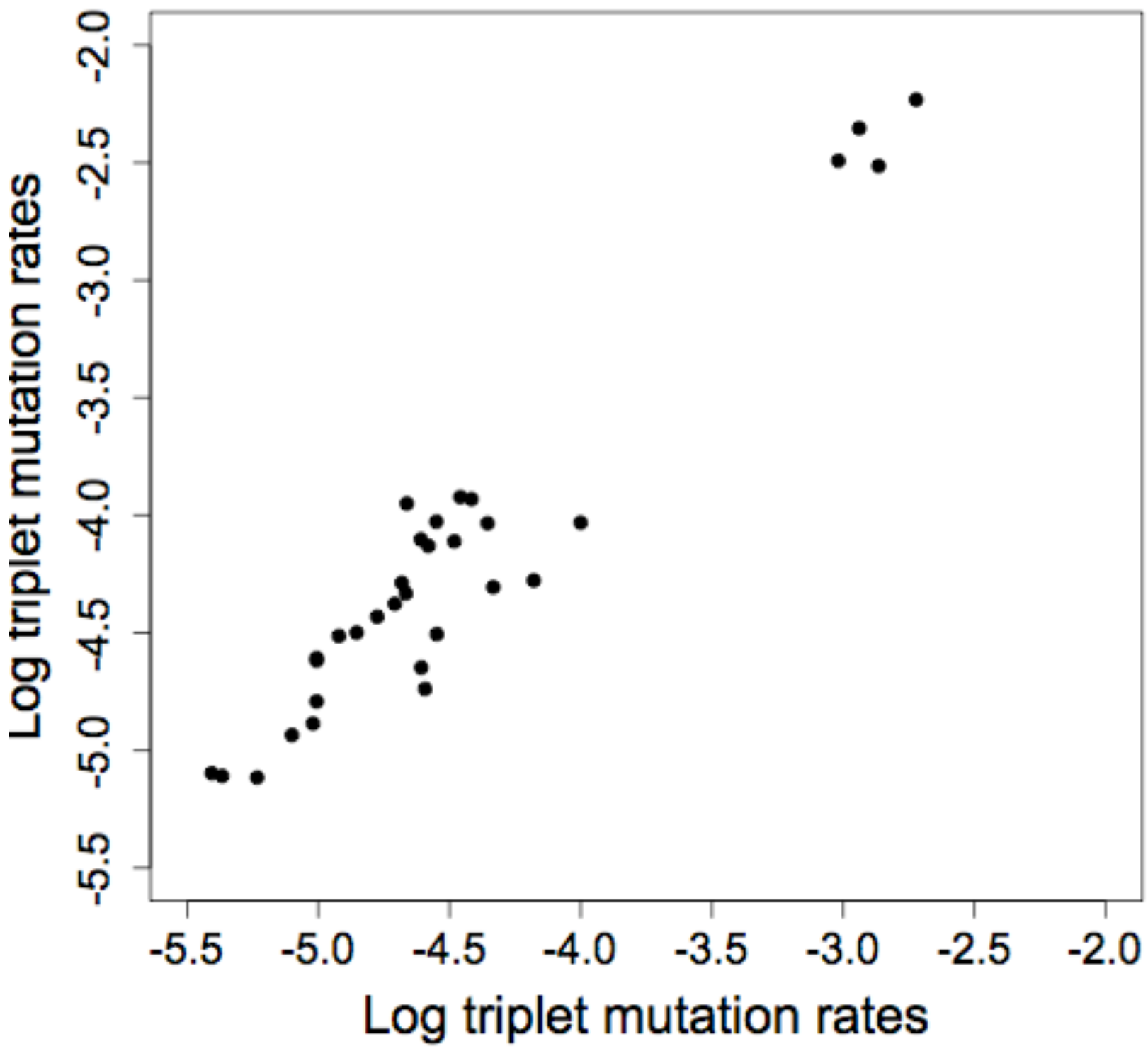
Log triplet mutation rates
-5.5
-5.0
-4.5
-4.0
-3.5
-3.0
-2.5
-2.0
Log triplet mutation rates
