# Supplementary material for: Cryptic Variation in the Human Mutation Rate
Source: PLoS Biol. 2009 Feb 3;7(2):e1000027. doi: 10.1371/journal.pbio.1000027 (PMC2634788; doi:10.1371/journal.pbio.1000027)
Supplement: Supplementary file 4 [file pbio.1000027.sd001.doc]

**Cryptic Variation in the Human Mutation Rate**

Alan Hodgkinson

Emmanuel Ladoukakis

Adam Eyre-Walker

**Supplementary Information**

In this supplementary information we derive the equations for the two state model of hypermutation. We also provide a number of supplementary tables and figures referred to in the main text.

Let us assume that a proportion, α, of sites are hypermutable and that they mutate at a rate β, whereas normal sites mutate at a rate of β^*^. Let us assume that hypermutable sites are always hypermutable. The average rate of mutation in the sequence is therefore

$\overline{m}=\text{αβ}+(1-\alpha)\beta$	(1)

If we arbitrarily set the average rate of mutation to one then we can express β^*^ in terms of α and β.

$\beta=\frac{1-\text{αβ}}{1-\alpha}$	(2)

Let the average probability of detecting a SNP in humans and chimpanzees be μ_h_ and μ_c_ respectively, then the expected number of coincident SNPs is

$P=\text{αm}_{h}m_{c}\beta^{2}+(1-\alpha)m_{h}m_{c}\beta^{2}$	(3)

If there is no variation in the mutation rate then the expected number of coincident SNPs is

$P_{0}=m_{h}m_{c}$	(4)

So the observed number of SNPs over the number expected with no variation is

$Z=\frac{P}{P_{0}}=\text{αβ}^{2}+(1-\alpha)\beta^{2}$	(5)

The expected excess of coincident SNPs therefore depends upon the proportion of sites that are hypermutable and the ratio β/β^*^. The observed excess of coincident SNPs at non-CpG sites is 1.98; we therefore set α to a range of values and solved equation 5 for β/β^*^. The results are shown in supplementary table 4.

**Table S1.** The number of times a particular SNP in humans is found opposite a particular SNP in chimpanzees, and the observed over expected ratio excluding CpG sites.

Note, some of the observed values are greater than when we included CpG dinucleotides. This is because we re-ran the analysis and when a chimp SNP had matched multiple human sequences we chose a sequence in which the human SNP was not involved in a CpG. Ratios are omitted when the expected value was less than 20.

**Table S2.** The observed and expected numbers of coincident SNPs in the alignments with high or low GC content.

Standard errors are given for the ratio.

**Table S3.** The observed and expected number of coincident SNPs from simulations run with different levels of CpG hypermutation and divergence.

**Table S4.** The relative rates of mutation at normal and hypermutable sites in the two-rate model.

**Figure S1.** The number of human SNPs at each site of the human-chimpanzee alignments used in the analysis excluding CpG sites.

The slight deficit of human SNPs adjacent to the chimpanzee is due to the fact that the adjacent sites are more likely to be inferred to be within a CpG because the chimp SNP might contain either C or G. For example, if the human SNP at +1 is G/A and the chimp SNP is C/G this would be called a potential CpG site and excluded.

**Figure S2.** The rate of mutation for each triplet and its reverse complement.

(a) all genes and (b) genes expressed in the testes.

**Figure S3.** The rate of mutation for each triplet in the GC-rich alignments (x-axis) versus the rate of mutation in the GC-poor alignments (y-axis).
