# Supplementary material for: Cryptic Variation in the Human Mutation Rate
Source: PLoS Biol. 2009 Feb 3;7(2):e1000027. doi: 10.1371/journal.pbio.1000027 (PMC2634788; doi:10.1371/journal.pbio.1000027)
Supplement: Supplementary file 5 [file pbio.1000027.st001.doc]

|  |  | Chimpanzee | | | | | |
| --- | --- | --- | --- | --- | --- | --- | --- |
|  |  | C/T | G/A | C/A | G/T | C/G | A/T |
| Observed | |  |  |  |  |  |  |
|  | C/T | 1120 | 1 | 103 | 46 | 101 | 53 |
|  | G/A | 3 | 1105 | 53 | 99 | 77 | 60 |
|  | C/A | 117 | 54 | 294 | 0 | 46 | 23 |
|  | G/T | 57 | 138 | 0 | 308 | 35 | 13 |
|  | C/G | 79 | 89 | 34 | 30 | 167 | 1 |
|  | A/T | 57 | 56 | 28 | 20 | 0 | 561 |
| Observed/expected | |  |  |  |  |  |  |
|  | C/T | 2.23 | - | 0.99 | 1.12 | 1.23 | 1.04 |
|  | G/A | - | 2.17 | 1.30 | 0.96 | 0.91 | 1.20 |
|  | C/A | 1.13 | 1.15 | 5.12 | - | 1.51 | 1.30 |
|  | G/T | 1.25 | 1.34 | - | 5.38 | 1.14 | 0.71 |
|  | C/G | 1.05 | 1.17 | 1.23 | 1.09 | 2.78 | - |
|  | A/T | 1.07 | 1.07 | 1.69 | 1.16 | - | 14.90 |

**Table S1**. The pattern of coincident SNPs at non-CpG sites.

The table shows the number of times a particular SNP in humans is found opposite a particular SNP in chimpanzees, and the observed over expected ratio excluding CpG sites. Note, some of the observed values are greater than when we included CpG dinucleotides. This is because we re-ran the analysis and when a chimp SNP had matched multiple human sequences we chose a sequence in which the human SNP was not involved in a CpG. Ratios are omitted when the expected value was less than 20.
