# Supplementary material for: Cryptic Variation in the Human Mutation Rate
Source: PLoS Biol. 2009 Feb 3;7(2):e1000027. doi: 10.1371/journal.pbio.1000027 (PMC2634788; doi:10.1371/journal.pbio.1000027)
Supplement: Supplementary file 6 [file pbio.1000027.st002.doc]

|  | All sites | | | Non-CpG sites | | |
| --- | --- | --- | --- | --- | --- | --- |
| GC content | Observed | Expected | Ratio  (SE) | Observed | Expected | Ratio  (SE) |
| Lower quartile | 2793 | 1420 | 1.97 (0.04) | 1633 | 752 | 2.17 (0.05) |
| Upper quartile | 2890 | 1862 | 1.55 (0.03) | 606 | 367 | 1.65 (0.07) |

**Table S2.** The observed and expected numbers of coincident SNPs in the alignments with high or low GC content.
