# Supplementary material for: Cryptic Variation in the Human Mutation Rate
Source: PLoS Biol. 2009 Feb 3;7(2):e1000027. doi: 10.1371/journal.pbio.1000027 (PMC2634788; doi:10.1371/journal.pbio.1000027)
Supplement: Supplementary file 7 [file pbio.1000027.st003.doc]

|  |  | All sites | | | Non CpG sites | | |
| --- | --- | --- | --- | --- | --- | --- | --- |
| Div (%) | CpG rate | Obs | Exp | Ratio | Obs | Exp | Ratio |
| 0 | 0 | 57 | 62 | 0.92 | 41 | 50 | 0.82 |
| 0 | 10x | 126 | 118 | 1.07 | 27 | 35 | 0.77 |
| 0 | 15x | 148 | 116 | 1.28 | 26 | 27 | 0.96 |
| 0 | 20x | 247 | 200 | 1.24 | 22 | 25 | 0.88 |
| 1 | 0 | 52 | 59 | 0.88 | 43 | 48 | 0.90 |
| 1 | 10x | 105 | 101 | 1.04 | 29 | 33 | 0.88 |
| 1 | 15x | 159 | 136 | 1.17 | 30 | 29 | 1.03 |
| 1 | 20x | 210 | 162 | 1.30 | 31 | 27 | 1.20 |
| 2 | 0 | 44 | 55 | 0.80 | 28 | 44 | 0.64 |
| 2 | 10x | 94 | 87 | 1.08 | 20 | 31 | 0.65 |
| 2 | 15x | 120 | 107 | 1.12 | 22 | 28 | 0.79 |
| 2 | 20x | 162 | 137 | 1.18 | 30 | 26 | 1.15 |

**Table S3.** The observed and expected number of coincident SNPs from simulations run with different levels of CpG hypermutation and divergence.
