# Supplementary material for: Cryptic Variation in the Human Mutation Rate
Source: PLoS Biol. 2009 Feb 3;7(2):e1000027. doi: 10.1371/journal.pbio.1000027 (PMC2634788; doi:10.1371/journal.pbio.1000027)
Supplement: Supplementary file 8 [file pbio.1000027.st004.doc]

| α | β/β^*^ |
| --- | --- |
| 0.001 | 33 |
| 0.002 | 24 |
| 0.01 | 12 |
| 0.02 | 9.2 |
| 0.1 | 5.9 |
| 0.2 | 5.9 |

**Table S4.** The relative rates of mutation at normal and hypermutable sites in the two-rate model.
